# Reproductive and genital health and risk of cervical human papillomavirus infection: results from the Ludwig-McGill cohort study

**DOI:** 10.1186/s12879-016-1446-x

**Published:** 2016-03-08

**Authors:** Eileen Shaw, Agnihotram V. Ramanakumar, Mariam El-Zein, Flavia R. Silva, Lenice Galan, Maria L. Baggio, Luisa L. Villa, Eduardo L. Franco, for the Ludwig-McGill Cohort Study

**Affiliations:** Division of Cancer Epidemiology, Department of Oncology, McGill University, Montreal, Canada; Department of Epidemiology, Biostatistics and Occupational Health, McGill University, 546 Pine Avenue West, Montreal, QC H2W 1S6 Canada; Ludwig Institute for Cancer Research, São Paulo, Brazil; Molecular Biology Laboratory, Centre of Translational Oncology, Instituto do Câncer do Estado de São Paulo, São Paulo, Brazil

**Keywords:** Human papillomavirus, HPV, Cervical cancer, Reproductive health, Genital hygiene, Determinants

## Abstract

**Background:**

There are inconsistencies in the literature on reproductive and genital health determinants of human papillomavirus (HPV) infection, the primary cause of cervical cancer. We examined these factors in the Ludwig-McGill Cohort Study, a longitudinal, repeated-measurements investigation on the natural history of HPV infection.

**Methods:**

We analyzed a cohort subset of 1867 women with one complete year of follow-up. We calculated odds ratios (OR) and 95 % confidence intervals (CI) for reproductive and genital health characteristics from questionnaire and laboratory data in relation to 1-year period prevalence of HPV infection. Two outcomes were measured; the first based on phylogenetic grouping of HPV types based on tissue tropism and oncogenicity (Alphapapillomavirus Subgenus 1: species 1, 8, 10 and 13; Subgenus 2: species 5, 6, 7, 9, 11; Subgenus 3: species 3, 4 and 14) and the second based on transient or persistent HPV infections.

**Results:**

Lifetime (Subgenus 3 OR = 2.00, CI: 1.23–3.24) and current (Subgenus 3 OR = 2.00, CI: 1.15–3.47) condom use and use of contraceptive injections (Subgenus 1 OR = 1.96, CI: 1.22–3.16, Subgenus 2 OR = 1.34, CI: 1.00–1.79) were associated with increased risk of HPV infection. Intrauterine device use was protective (Subgenus 1 OR = 0.48, CI: 0.30–0.75, Subgenus 2 OR = 0.78, CI: 0.62–0.98). These factors were not associated with persistence of HPV infection. Tampon use, previous gynecologic infections and cervical inflammation were associated with an overall increased risk of HPV infection.

**Conclusions:**

Cervical HPV infection was associated with reproductive and genital health factors. Further studies are necessary to confirm the low to moderate associations observed.

**Electronic supplementary material:**

The online version of this article (doi:10.1186/s12879-016-1446-x) contains supplementary material, which is available to authorized users.

## Background

Cervical cancer is the fourth most common cancer in women worldwide [1]. Despite recent advances in human papillomavirus (HPV) vaccines and screening programs, cervical cancer remains a large global health burden. HPV is a necessary cause of cervical cancer, but it is not sufficient. Therefore, identification of risk factors of HPV infection and cervical cancer may inform preventive strategies. Outside of sexual behavior characteristics and parity [2–4], there is a lack of consensus in the literature on determinants of HPV infection, particularly with respect to reproductive and genital health.

Condom use has been inconsistently associated with a decreased risk of HPV infection [5, 6]. Use of oral contraceptives (OCs) is associated with cervical carcinogenesis [7, 8], while use of intrauterine devices (IUDs) [9] and tubal sterilization [10] seems protective. However, evidence of a relationship between these factors and risk of HPV infection remains equivocal as there have been very few studies done in this field [10, 11]. An elevated risk of HPV infection is associated with other infections such as *Chlamydia trachomatis* [12, 13], human immunodeficiency virus (HIV) [14, 15], and bacterial vaginosis (BV) [16]. Despite these trends, inconsistencies remain concerning the magnitude and direction of effects, with a paucity of information on other factors.

This study focuses on the role of reproductive and genital health on cervical HPV infections using data from a large longitudinal study conducted to investigate the natural history of HPV infection.

## Methods

### Study design and participants

The design and methods of the Ludwig-McGill Cohort Study have been previously described [17]. The study enrolled women from São Paulo, Brazil, a high-risk area for cervical cancer. Briefly, women were recruited from a maternal and child health program for low-income families between 1993 and 1997. Eligible women must have: 1) been 18–60 years old 2) been permanent residents of São Paulo 3) had no intention of becoming pregnant over the next year 4) had an intact uterus without referral for hysterectomy 5) had no treatment for cervical disease within 6 months previous to enrolment and 6) reported no use of vaginal medication in the 2 days prior to enrolment. Signed, informed consent for participation in the study was obtained from all women. Follow-up of women occurred every 4 months in the first year and twice per year afterwards. The study was approved by the institutional review boards and ethical committees of McGill University, University of Toronto, the Ludwig Institute for Cancer Research and the Maternidade Escola Vila Nova Cachoeirinha clinic.

### Data collection and procedures

Questionnaires were administered by one of two study nurses (MLB, LG) at each of the four visits in the first year and once per year afterwards. Baseline questionnaires collected detailed information on sexual and reproductive histories, and behavioural characteristics including smoking and hygiene habits. Cervical specimens were taken at each visit for conventional Pap cytology and molecular HPV testing. Ectocervical and endocervical cells were collected using an Accelon biosampler (Medscand Inc., Hollywood, FL). Pap smears were read and graded at the Jewish General Hospital (Montreal, Canada).

Presence of HPV DNA was detected using polymerase chain reaction to amplify a highly conserved segment of the L1 viral gene using PGMY consensus primers [18, 19]. Typing of the amplified product was performed by hybridization with individual oligonucleotide probes, and by restriction fragment-length polymorphism analysis to identify 40 different mucosal HPV types. We grouped types according to Alphapapillomavirus species clusters that exhibit comparable tissue tropism and biological behavior concerning cancer risk [20–23]. Subgenus 1 included HPV types 6, 11, 32, 40, 42, 44 and 54, from species α1, α8, α10 and α13. Subgenus 2 included HPV types 16, 18, 26, 31, 33, 34, 35, 39, 45, 51, 52, 53, 56, 58, 59, 66, 67, 68, 69, 70, 73 and 82, from species α5, α6, α7, α9 and α11. Subgenus 3 included HPV types 57, 61, 62, 71, 72, 81, 83, 84 and 89, from species α3, α4 and α14. Subgenus 1 and Subgenus 3 HPV types are part of subgenera whose members are not carcinogenic, whereas types in Subgenus 2 are mostly carcinogenic. Types in all three subgenus groups exhibit strong genital tropism but types from Subgenus 3 are mostly commensal agents that infect the vagina but produce no clinically identifiable lesions [20–23].

### Exposure measurements and study outcomes

Aside from questions with predetermined answer choices, responses to open-ended questions were grouped into categorical variables, including comments disclosed to the administering nurses, but not explicitly asked (Additional file 1: Table S1). Some exposures were classified as “former”, indicating lifetime use and “current”, indicating use between the first and second visit as use between visits remained relatively constant over the first year of follow-up. We also grouped qualitative cytology results, which included reports on inflammation and bacterial or fungal infections into categories relating to cervical health conditions.

We used two HPV outcome variables. First, we considered 1-year period prevalence for infections from the above three phylogenetically-defined subgenus groups by taking into account HPV types detected cumulatively over the first four visits in the first year (enrolment and months 4, 8 and 12). Second, we categorized HPV infections into mutually exclusive transient or persistent infections in the first year. Transient HPV infections were those involving one positive HPV test result followed by two subsequent negative results. Persistent infections were two or more positive HPV test results of the same HPV group, with no more than one negative result between two positive test results. HPV test results that did not fall into these definitions were excluded.

### Statistical analysis

We estimated odds ratios (ORs) and 95 % confidence intervals (CIs) for HPV outcomes using logistic regression models, adjusting for *a priori* (baseline age and lifetime number of sexual partners) and empirical confounders. The latter, identified using a 5 % change-in-estimate strategy, included race, marital status, education, age at first intercourse, age at menarche, number of pregnancies, smoking, alcohol drinking and years since last Pap smear.

For the analysis of 1-year period prevalence, we compared women with a specific group infection (Subgenus 1 = 108, Subgenus 2 = 495, Subgenus 3 = 131) to a floating control group of those who did not have that group infection. We called this the non-restricted analysis. Since concurrent and cumulative multiple-type infections were common in the cohort, we conducted separate analyses restricted to women who only harboured infections with HPV types from a single phylogenetic group (Subgenus 1 = 41, Subgenus 2 = 377, Subgenus 3 = 57). A fixed control group that included only HPV-negative women (*n* = 1267) was used in these restricted analyses. The restricted analyses, while compromising on precision, were intended to increase the validity of observed associations and were thus used to confirm associations seen in the non-restricted estimates. Adjusted ORs were calculated using the non-restricted crude estimate. All data were analyzed using Stata 12.0 (StataCorp, College Station, TX, USA).

## Results

**Fig. 1:**
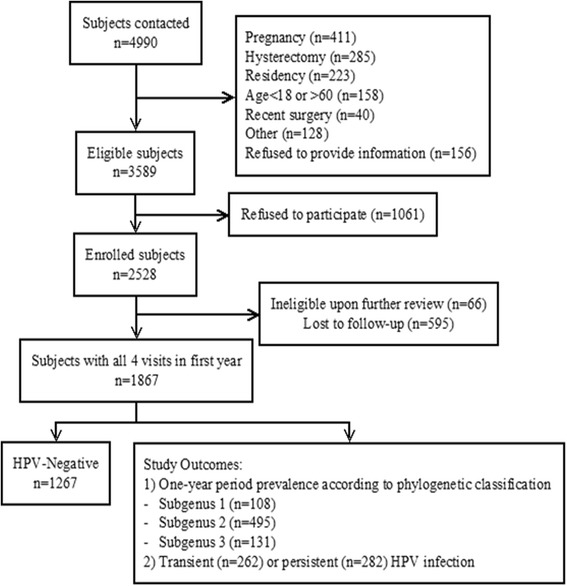
Flow chart of the selection of analytical study sample population. The study sample included a subset of 1867 women with one complete year of follow-up from the Ludwig-McGill Cohort Study population. Two outcomes were considered. The first was based on phylogenetic grouping of HPV types based on tissue tropism and oncogenicity (Alphapapillomavirus Subgenus 1, 2 and 3). The second outcome was based on transient or persistent HPV infections

Of the 3589 eligible women, 2528 participants were enrolled in the Ludwig-McGill cohort study (Fig. 1), with an average follow-up time of 7 years. This analysis was restricted to 1867 women who had completed all four visits within the first year, representing 76 % of those enrolled in the study. Of these, 1267 women were HPV-negative and 600 women tested positive for any HPV infection on at least one of the four visits. Due to multiple infections in some women, there were a total of 734 type-specific HPV infections. For the second outcome indicator, there were 262 transient HPV infections, 282 persistent infections and 56 women were excluded from this analysis. At baseline, the mean age of participants was 32.9 (±8.7) years, and the mean number of lifetime sexual partners and pregnancies were 3.9 and 3.5, respectively. Only 19 % of women had completed high school or further education. Characteristics of this study population according to outcome variables are described in Additional file 1: Table S2.

Table 1 presents associations for reproductive and genital health factors with the first outcome of phylogenetic grouping of HPV types (Additional file 1: Table S3 refers to the restricted subsets). Results from the restricted crude analyses tended to reflect those of the non-restricted crude and adjusted analyses apart from the expected loss in precision in the restricted estimates. Table 2 presents these associations for the second outcome of transient and persistent infections.

**Table 1:** Associations between reproductive health and vaginal hygiene factors and the 1-year period prevalence of HPV infection by phylogenetic group in the Ludwig-McGill cohort study

Variable	Subgenus 1^a^ (*n* = 108)		Subgenus 2^a^ (*n* = 495)		Subgenus 3^a^ (*n* = 131)	
	OR^b^	95 % CI	OR^b^	95 % CI	OR^b^	95 % CI
Reproductive Health Factors						
Condom Use						
Former vs. Never	1.28	0.79, 2.07	0.90	0.71, 1.15	2.00	1.23, 3.24
Current vs. Never	1.43	0.83, 2.48	1.09	0.82, 1.45	2.00	1.15, 3.47
Oral Contraceptives						
< 6 years vs. Never	1.25	0.66, 1.38	0.98	0.71, 1.35	1.40	0.83, 2.37
6+ years vs. Never	1.23	0.60, 2.53	1.00	0.70, 1.43	0.62	0.32, 1.19
Intrauterine Device						
Yes vs. No	0.48	0.30, 0.75	0.78	0.62, 0.98	0.73	0.49, 1.08
Tubal Sterilization						
Yes vs. No	1.24	0.65, 1.36	0.84	0.59, 1.21	0.65	0.33, 1.30
Contraceptive Injection						
Yes vs. No	1.96	1.22, 3.16	1.34	1.00, 1.79	1.34	0.83, 2.17
Natural Products						
Yes vs. No	-	-	0.37	0.13, 1.05	0.74	0.17, 3.20
Vaginal Products						
Yes vs. No	-	-	0.82	0.37, 1.81	0.32	0.04, 2.40
Genital Health and Hygiene Factors						
Menstrual Cloth						
Yes vs. No	0.54	0.32, 0.91	0.93	0.74, 1.18	1.02	0.68, 1.53
Hygienic Tampon						
Yes vs. No	1.49	0.87, 2.54	1.33	0.98, 1.81	1.30	0.79, 2.14
Douching						
Frequent vs. Infrequent	1.20	0.53, 2.69	1.29	0.83, 2.00	0.11	0.02, 0.81
Douching Products						
Natural vs. None	0.96	0.60, 1.54	0.92	0.72, 1.17	0.90	0.57, 1.41
Medical vs. None	1.09	0.23, 5.15	0.97	0.46, 2.06	2.75	0.98, 7.73
Unknown vs. None	1.18	0.27, 5.22	0.51	0.21, 1.21	2.92	1.20, 7.12
Genital Discomfort						
Yes vs. No	0.96	0.64, 1.45	0.96	0.78, 1.20	0.89	0.60, 1.31
Recent Discomfort						
Yes vs. No	0.99	0.65, 1.50	1.11	0.89, 1.38	1.22	0.84, 1.75
Pain or Bleeding						
Yes vs. No	2.83	0.59, 13.51	1.14	0.34, 3.75	0.91	0.12, 7.00
Gynecologic Products						
Antibiotic						
Former vs. Never	3.86	0.86, 17.34	0.83	0.51, 1.33	1.43	0.52, 3.97
Current vs. Never	5.95	1.14, 30.97	0.97	0.53, 1.79	1.84	0.56, 6.02
Antifungal						
Former vs. Never	5.14	1.17, 22.52	1.10	0.70, 1.75	1.85	0.68, 5.02
Current vs. Never	1.64	0.22, 12.24	1.06	0.56, 2.02	1.74	0.49, 6.11
Abrasion						
Former vs. Never	-	-	1.68	0.40, 7.72	-	-
Current vs. Never	-	-	1.10	0.08, 16.23	-	-
External Products						
Former vs. Never	0.75	0.20, 2.84	-	-	-	-
Current vs. Never	1.36	0.48, 3.89	-	-	-	-
Unknown Products						
Former vs. Never	2.82	0.64, 12.42	0.96	0.62, 1.49	2.21	0.81, 6.01
Current vs. Never	3.44	0.63, 18.68	1.10	0.60, 2.03	2.82	0.83, 9.53
Home-made Products						
Natural vs. None	1.22	0.78, 1.92	0.91	0.72, 1.15	0.98	0.66, 1.47
Medical vs. None	0.80	0.27, 2.40	1.26	0.77, 2.04	0.76	0.29, 2.02
Unknown vs. None	2.75	0.53, 14.15	1.33	0.45, 3.91	5.33	1.80, 15.82
Vaginal Health Characteristics						
Cervical Ectropion						
Yes vs. No	0.37	0.05, 2.77	0.89	0.42, 1.91	0.74	0.17, 3.10
Gynecologic Surgery						
Yes vs. No	2.05	1.11, 3.79	0.90	0.60, 1.37	0.89	0.42, 1.89
Gynecologic Treatment/Infection						
Yes vs. No	1.73	1.13, 2.65	1.17	0.92, 1.50	1.26	0.83, 1.89
Previous Gynecologic Infection						
HPV vs. None	3.87	2.09, 7.14	2.02	1.27, 3.19	1.21	0.54, 2.71
Non-HPV vs. None	1.33	0.81, 2.19	1.03	0.78, 1.34	1.27	0.82, 1.97
Cytology Observations						
Bacterial Infection						
Yes vs. No	1.28	0.63, 2.63	1.30	0.88, 1.91	1.31	0.68, 2.52
Fungal Infection						
Yes vs. No	1.25	0.61, 2.54	1.29	0.88, 1.87	1.48	0.83, 2.66
General Inflammation						
Yes vs. No	2.10	1.37, 3.22	1.53	1.21, 1.94	1.12	0.74, 1.69

^a^ Subgenus 1 (HPVs-6, 11, 32, 40, 42, 44, 54 and 55), Subgenus 2 (HPVs-16, 18, 26 31, 33–35, 39, 45, 51–53, 56, 58, 59, 66–70, 73 and 82) and Subgenus 3 (HPVs-57, 61, 62, 71, 72, 81, 83, 84 and 89) infections were determined based on the phylogenetic classification of HPV types

^b^ Odds ratios were adjusted for age, lifetime number of sexual partners and empirical confounders (identified using a 5 % change in estimate strategy) such as race, marital status, education, age at first intercourse, age at menarche, number of pregnancies, smoking, alcohol drinking and years since last Pap smear

**Table 2:** Associations between reproductive health and vaginal hygiene and transient or persistent HPV infections in the Ludwig-McGill cohort study

Variable	Transient HPV infection^a^		Persistent HPV infection^b^	
	OR^c^	95 % CI	OR^c^	95 % CI
Reproductive Health Factors				
Condom Use				
Former vs. Never	1.33	0.95, 1.86	1.06	0.77, 1.46
Current vs. Never	1.47	1.00, 2.16	1.24	0.86, 1.80
Oral Contraceptives				
< 6 years vs. Never	1.16	0.76, 1.75	1.02	0.69, 1.52
6+ years vs. Never	0.90	0.56, 1.46	1.14	0.73, 1.77
Intrauterine Device				
Yes vs. No	0.65	0.48, 0.87	0.77	0.57, 1.03
Tubal Sterilization				
Yes vs. No	0.78	0.47, 1.28	0.75	0.46, 1.23
Contraceptive Injection				
Yes vs. No	1.58	1.10, 2.26	1.31	0.91, 1.89
Natural Products				
Yes vs. No	0.17	0.02, 1.31	0.46	0.14, 1.55
Vaginal Products				
Yes vs. No	0.88	0.30, 2.56	0.76	0.26, 2.21
Genital Health and Hygiene Factors				
Menstrual Cloth				
Yes vs. No	1.00	0.74, 1.36	0.79	0.58, 1.06
Hygienic Tampon				
Yes vs. No	1.48	1.00, 2.20	1.42	0.96, 2.08
Douching				
Frequent vs. Infrequent	1.10	0.60, 2.00	1.12	0.62, 2.01
Douching Products				
Natural vs. None	0.86	0.62, 1.19	0.80	0.57, 1.12
Medical vs. None	0.82	0.28, 2.40	1.70	0.72, 4.02
Unknown vs. None	0.36	0.09, 1.51	1.03	0.37, 2.87
Genital Discomfort				
Yes vs. No	0.87	0.65, 1.18	0.89	0.67, 1.20
Recent Discomfort				
Yes vs. No	1.06	0.79, 1.41	1.22	0.93, 1.59
Pain or Bleeding				
Yes vs. No	0.61	0.07, 5.03	0.90	0.18, 4.48
Gynecologic Products				
Antibiotic				
Former vs. Never	1.00	0.53, 1.89	2.09	1.10, 3.98
Current vs. Never	0.89	0.37, 2.15	2.50	1.23, 5.08
Antifungal				
Former vs. Never	1.74	0.94, 3.20	1.51	0.81, 2.82
Current vs. Never	1.52	0.67, 3.45	1.80	0.78, 4.13
Abrasion				
Former vs. Never	4.17	0.49, 35.27	2.47	0.40, 15.26
Current vs. Never	5.40	0.26, 113.29	-	-
External Products				
Former vs. Never	1.46	0.18, 11.99	0.91	0.08, 10.66
Current vs. Never	6.43	0.65, 63.70	-	-
Unknown Products				
Former vs. Never	1.03	0.58, 1.84	1.14	0.65, 2.02
Current vs. Never	1.31	0.62, 2.81	1.93	0.95, 3.93
Home-made Products				
Natural vs. None	1.00	0.74, 1.37	0.93	0.69, 1.26
Medical vs. None	0.97	0.47, 1.98	0.77	0.38, 1.56
Unknown vs. None	1.17	0.24, 5.73	3.20	1.00, 10.24
Vaginal Health Characteristics				
Cervical Ectropion				
Yes vs. No	1.01	0.41, 2.52	0.81	0.30, 2.19
Gynecologic Surgery				
Yes vs. No	1.22	0.72, 2.04	1.39	0.86, 2.25
Gynecologic Infection/Treatment				
Yes vs. No	0.90	0.65, 1.24	1.14	0.84, 1.54
Previous Gynecologic Infection				
HPV vs. None	3.23	1.91, 5.47	2.46	1.40, 4.31
Non-HPV vs. None	0.95	0.66, 1.37	1.15	0.82, 1.61
Cytology Observations				
Bacterial Infection				
Yes vs. No	1.16	0.69, 1.95	1.45	0.91, 2.31
Fungal Infection				
Yes vs. No	1.05	0.63, 1.75	1.24	0.77, 2.01
General Inflammation				
Yes vs. No	1.73	1.27, 2.35	1.62	1.20, 2.19

^a^ Transient HPV infections were defined as one positive test result in the first four visits, followed by two subsequent negative results

^b^ Persistent HPV infections were defined as two or more positive HPV test results over the first four visits, with no more than one negative result between two positive test results

^c^ Odds ratios were adjusted for age, lifetime number of sexual partners and empirical confounders (identified using a 5 % change in estimate strategy) such as race, marital status, education, age at first intercourse, age at menarche, number of pregnancies, smoking, alcohol drinking and years since last Pap smear

### Contraceptive methods

Both former (OR = 2.00, 95 % CI: 1.23–3.24) and current (OR = 2.00, 95 % CI: 1.15–3.47) condom use was significantly associated with an increased prevalence of Subgenus 3 HPV infections. Current condom use was also significantly associated with transient HPV infections (OR = 1.47, 95 % CI: 1.00–2.16). OC use was not significantly associated with any HPV outcome analyzed. Use of IUDs was protective across all HPV outcomes with significant effects in Subgenus 1 (OR = 0.48, 95 % CI: 0.30–0.75), Subgenus 2 (OR = 0.78, 95 % CI: 0.62–0.98) and transient (OR = 0.65, 95 % CI: 0.48–0.87) infections. Tubal sterilization had a predominantly protective, although insignificant, effect across all outcomes. Of the open-ended responses, use of contraceptive injections increased the prevalence of all HPV outcomes, with Subgenus 1 (OR = 1.96, 95 % CI: 1.22–3.16), Subgenus 2 (OR = 1.34, 95 % CI: 1.00–1.79) and transient (OR = 1.58, 95 % CI: 1.10–2.26) HPV infections being statistically significant. Natural and external products were not significantly associated with any HPV outcome, although there were not enough observations to calculate Subgenus 1 ORs.

### Vaginal hygiene

Menstrual cloth use had a protective effect for Subgenus 2, transient and persistent HPV infections with a significant effect on Subgenus 1 infections (OR = 0.54, 95 % CI: 0.32–0.91). Conversely, all point estimates for hygienic tampon use were above the null with Subgenus 2 infections approaching borderline significance and transient infections being statistically significant (OR = 1.48, 95 % CI: 1.00–2.20). Frequent douching was not associated with most HPV outcomes, although there was a significantly protective effect on Subgenus 3 infections (OR = 0.11, 95 % CI: 0.02–0.81). There was also no effect of the types of douching products used, with the exception of “unknown” products and Subgenus 3 HPV infections (OR = 2.92, 95 % CI: 1.20–7.12). Lastly, genital discomfort, recent discomfort and reported pain/bleeding were not associated with any HPV infection outcome.

### Gynecologic products

In general, no significant associations or trends were observed with the use of gynecologic products and some categories lacked observations. Of note, both former (OR = 2.09, 95 % CI: 1.10–3.98) and current (OR = 2.50, 95 % CI: 1.23–5.08) antibiotic use were associated with persistent HPV infections, while current antibiotic use (OR = 5.95, 95 % CI: 1.14–30.97) and former antifungal use (OR = 5.14, 95 % CI: 1.17–22.52) were significantly associated with Subgenus 1 infections. No significant trends were observed with the use of homemade gynecologic products, other than use of “unknown” homemade products and Subgenus 3 infections (OR = 5.33, 95 % CI: 1.80–15.85) and persistent HPV infections (OR = 3.20, 95 % CI: 1.00–10.24). Due to low numbers of women using these products, the CIs were very wide, indicating low precision in the estimates.

### Vaginal health

From information volunteered by the participant and not explicitly asked in questionnaires, there were no significant associations between cervical ectropion and any HPV infection outcome. A self-report of gynecologic surgery was associated with an increased prevalence of Subgenus 1 HPV infections (OR = 2.05, 95 % CI: 1.11–3.79). Information on previous gynecologic infections or treatments volunteered by the participant was also associated with a significant increase in prevalence of Subgenus 1 HPV infections (OR = 1.73, 95 % CI: 1.13–2.65) with no association with other HPV outcomes. When previous history of gynecologic infections was explicitly asked, there were no significant associations between non-HPV-related infections and any HPV infection outcome. There was, however, a significant increase between previous HPV-related infections and Subgenus 1 (OR = 3.87, 95 % CI: 2.09–7.14), Subgenus 2 (OR = 2.02, 95 % CI: 1.27–3.19), transient (OR = 3.23, 95 % CI: 1.91–5.47) and persistent (OR = 2.46, 95 % CI: 1.40–4.31) HPV infections. Using qualitative notes from the cytology reports, there were no significant associations with bacterial or fungal infections and any HPV infection. General inflammation was positively associated with Subgenus 1 (OR = 2.10, 95 % CI: 1.37–3.22), Subgenus 2 (OR = 1.53, 95 % CI: 1.21–1.94), transient (OR = 1.73, 95 % CI: 1.27–2.35) and persistent (OR = 1.62, 95 % CI: 1.20–2.19) HPV infections.

## Discussion

This study provides novel insights on reproductive health and vaginal hygiene factors in HPV infection. While some of these factors seemed to be associated with adverse or protective effects regarding cervical HPV infection, they largely did not play an appreciable role on the risk of infection in the Ludwig-McGill cohort study population.

Previous studies [5] have demonstrated various directions of effect in condom use and HPV infection, despite the underlying hypothesis of condoms being protective. In our study, condom use seemed to be a risk marker for Subgenus 3 HPV infections, which have tropism for vaginal tissue. We did not measure frequency of condom use, thus it is possible that women who reported condom use, but did not consistently use them during sexual intercourse, could still be exposed to HPV. Sexual behaviour could play a role in these associations. Upon stratifying by marital status and number of lifetime sexual partners, the risk-marker effect of condom use was stronger in women who were not in stable relationships (single, separated or widowed) and in women with three or more lifetime sexual partners at baseline (data not shown).

IUD use was associated with an overall protective effect, consistent with other studies [24, 25]. While our study did not measure differences in use between copper and hormonal IUDs, which release progestin to prevent fertilization, the protective effect on Subgenus 2 HPV infections could have resulted from a low-level immune response triggered by IUDs in the uterine endometrium and cervix. There was a general protective trend with tubal sterilization, which could be associated with behavioural factors, as tubal sterilization was more common in women in stable relationships. Moreover, due to the irreversibility of the procedure, it is more common in older women. Thus, there could be an effect of age in this interaction as protective effect was predominantly seen in women over 30 years of age (data not shown). It is also possible that the protective effects for IUD use and tubal ligation may have resulted from low viral transmission in low-risk partnerships. Although we controlled for empirical confounders, these were based on female interviews. We did not have behavior data on male partners, which would have allowed us to verify the low-risk partnership hypothesis.

Contraceptive injection use was associated with an increased prevalence of Subgenus 1, Subgenus 2 and transient HPV infections. While previous studies have shown an increased association with cervical cancer [7, 26], few studies have investigated the role of contraceptive injections in HPV infection. There is precedence for a hormonal effect of progestogen-only contraceptives in increasing susceptibility or persistence of HPV infection (compared to combined OCs containing estrogen and progestin) [27, 28]. Sexual behaviour could also be a factor as contraceptive injection/Norplant use was more prevalent in women with three or more lifetime sexual partners (16.9 % vs. 12.9 %).

Use of menstrual cloths had a slightly protective effect on HPV infections, while use of hygienic tampons had an adverse effect. Use of menstrual cloths has not previously been studied in association with HPV infection and there does not appear to be a biological mechanism for this protective effect. Very limited evidence exists for an association between tampon use and risk of HPV infection. Some studies show no association [29], while some report a positive association between tampon use and high-risk HPV infection [30]. We speculate that tampon use can lead to dryness and irritation in the vagina and cervix [31], thereby increasing susceptibility to HPV infections through possible tearing or microabrasions.

Gynecologic product use served as a proxy for vaginal health conditions. Our results suggest that bacterial infections and use of products to control these infections may play a role in increasing susceptibility to HPV infection. BV has been positively associated with HPV infections [16], which is biologically plausible. A lack of protective lactobacilli in the vaginal flora during BV can increase susceptibility to infections, such as *Chlamydia trachomatis*, *Neisseria gonorrhoeae*, HSV-1 and 2, HIV [32] and possibly HPV infections.

Previous gynecologic infections were associated with increased prevalence of Subgenus 1 infections, which we hypothesize to be related to BV. Self-reports of HPV-related infections were significantly associated with all outcomes, except for Subgenus 3 infections. Women who reported a previous HPV-related infection could have an increased risk of re-infection due to sexual behaviours or, just as plausibly, redetection of a latent HPV infection. General inflammation noted in cytology results was significantly associated with all HPV outcomes other than Subgenus 3 infections. A possible link between inflammation and cervical carcinogenesis might exist. There are indications that chronic inflammation or inflammation from other infections may play a role in increasing risk of HPV infection [13], possibly explaining this association.

Surprisingly, cervical ectropion was not associated with any HPV outcome. The location of the transformation zone can affect potential exposure to HPV infection and risk of cervical cancer [22]. Our results must be taken with reservation as this was not explicitly asked in the questionnaire. Due to mild symptoms, if any at all, women may not be aware of the condition unless diagnosed by gynecological examination, leading to potential misclassification. We found a positive association between gynecologic surgery and Subgenus 1 HPV infections. Most surgeries were dilation and curettage, a procedure sometimes used to further diagnose a suspected HPV infection in the endocervix. This might explain the observed positive association, as some cases of gynecologic surgery may be in women who were already HPV-infected.

In restricting our analysis to only women with one full year of follow-up, we may have introduced a selection bias. However, we believe that this enhanced internal validity and efficiency in defining the HPV outcome variables. Furthermore, losses to follow-up in the first year did not play a significant role as preliminary analyses of the non-restricted data yielded similar results. We classified type-specific HPV outcomes into groups based on phylogenetic relatedness to guide the statistical search for predictors on the basis of viral behavior and tropism. We were unable to study individual type-specific infections due to lack of precision.

Several limitations need to be underscored. Due to the low-income setting of the study clinics where patients were recruited from, the study population was very specific, with respect to cultural and social norms. Secondly, outside of qualitative observations from cytology reports, exposure measurements were based on self-reported responses. As this study pertains to sexual histories and behaviour, there is a possible social desirability bias where women, especially those with riskier sexual behaviours, would tend to answer questions on sensitive topics in a socially acceptable manner. Additionally, poor recall, especially on lifetime exposures, might be at play. While we anticipate that any possible exposure misclassification would be non-differential to outcome status, women with a previous history of gynecologic infections may be more conscious of the products they have previously used. In addition, the Ludwig-McGill cohort study was not designed to investigate measures of vaginal health and hygiene as a primary objective. This post hoc analysis considered exposure measures that predominantly served as proxies for vaginal conditions. Because several of these conditions are often asymptomatic or characterized by mild symptoms, women may not have been aware of these conditions. Lastly, there could still be residual confounding due to unmeasured variables, particularly related to male behaviors.

## Conclusions

This study presents detailed analyses on the effects of vaginal health and hygiene factors, some of which seemed to be associated with risk of HPV infection differentially by subgenus grouping based on propensity to colonize different lower genital tract tissue subsites. Further research using hypothesis-driven designs with more accurate measures of exposure is necessary to examine the observed associations.

## Additional file

Additional file 1: Table S1. Open-ended questionnaire variable category designations from the first four patient visits, including qualitative observations from cytology results in the Ludwig-McGill Cohort Study. **Table S2.** Characteristics of the Ludwig-McGill Cohort Study population by HPV infection according to phylogenetic group and transient or persistent infections. **Table S3.** Crude and restricted crude (CrudeR) odds ratios of associations between reproductive health and vaginal hygiene and the 1-year period prevalence of HPV infection according to phylogenetic group in the Ludwig-McGill Cohort Study. (DOC 331 kb)
