# Supplementary material for: Reproductive and genital health and risk of cervical human papillomavirus infection: results from the Ludwig-McGill cohort study
Source: BMC Infect Dis. 2016 Mar 8;16:116. doi: 10.1186/s12879-016-1446-x (PMC4782350; doi:10.1186/s12879-016-1446-x)
Supplement: Additional file 1: Table S1. — Open-ended questionnaire variable category designations from the first four patient visits, including qualitative observations from cytology results in the Ludwig-McGill Cohort Study. Table S2. Characteristics of the Ludwig-McGill Cohort Study population by HPV infection according to phylogenetic group and transient or persistent infections. Table S3. Crude and restricted crude (CrudeR) odds ratios of associations between reproductive health and vaginal hygiene and the 1-year period prevalence of HPV infection according to phylogenetic group in the Ludwig-McGill Cohort Study. (DOC 331 kb) [file 12879_2016_1446_MOESM1_ESM.doc]

**Table S1.** Open-ended questionnaire variable category designations from the first four patient visits, including qualitative observations from cytology results in the Ludwig-McGill Cohort Study

| **Variable** | **Categories** | **Examples** |
| --- | --- | --- |
| **Gynecological products used** | Antibiotic | Talsutin, Gino-Pletil, Vagitine, Vagi-sulfa, Polygynax, Tricangine A |
|  | Antifungal | Mycostatin, Gyno-Daktarin, Ginedak, Nizoral, Nistatin |
|  | Chemical abrasives | Albocresil, Efurix, trichloroacetic acid |
|  | External use | Ginecoside, Lamisil, Calminex |
|  | Cauterization/other | Vaseline, glycerine, Novaderm, Colpotrofine |
|  | Unknown | Subject used a gynecologic product but does not remember/not know which product |
| **Homemade gynecological products used** | Natural | Tea, vinegar, crystal violet, herbs |
|  | Chemical/Medicinal | Flogo-rosa, Lysoform, Lucretin, Andolba |
|  | Unknown | See above |
| **Vaginal douching products** | Natural | Crystal violet, diluted permanganate |
|  | Chemical/Medicinal | Flogo-rosa, Lysoform, Lucretin |
|  | Unknown | See above |
| **Other contraceptives** | Injection/Norplant | Hormonal birth control injection (Depo-Provera) |
|  | Natural products | Tea, vinegar, vaginal douching |
|  | Vaginal products | Vaginal dressing, creams |
| **Voluntary comments** | Cervical Ectroption | N/A |
|  | Gynecological surgery | Dilation and Curettage, abortion |
|  | Pain or Bleeding | N/A |
|  | Gynecologic Infection/Treatment | Previous medications taken, symptoms such as discharge or smell |
| **Qualitative cytology observations^*^** | Bacterial infection | *Gardnerella vaginalis*, *Trichomonas vaginalis* |
|  | Fungal infection | *Candida albicans* |
|  | General inflammation | N/A |

N/A – Examples are not applicable

^*^Qualitative observations were noted by Dr. Alex Ferenczy’s laboratory at the Jewish General Hospital (Montreal, QC).

##### **Table S2.** Characteristics of the Ludwig-McGill Cohort Study population by HPV infection according to phylogenetic group and transient or persistent infections

|  | | |  |  |  | **Phylogenetic group^*^** | | |  | **Infection type^†^** | |
| --- | --- | --- | --- | --- | --- | --- | --- | --- | --- | --- | --- |
| **Variable** | | |  | **HPV-Negative**  (n=1267) |  | **Subgenus** **1**  (n=108) | **Subgenus** **2**  (n=495) | **Subgenus** **3**  (n=131) |  | **Transient**  (n=262) | **Persistent**  (n=282) |
|  | | |  | **n (%)** |  | **n (%)** | **n (%)** | **n (%)** |  | **n (%)** | **n (%)** |
| **Sociodemographic Characteristics** | | |  |  |  |  |  |  |  |  |  |
|  | Age | |  |  |  |  |  |  |  |  |  |
|  | | <30 |  | 436 (34.4) |  | 53 (49.1) | 235 (47.5) | 60 (45.8) |  | 134 (51.1) | 141 (50.0) |
|  | | 30+ |  | 831 (65.6) |  | 55 (50.9) | 260 (52.5) | 71 (54.2) |  | 128 (48.9) | 141 (50.0) |
|  | Race | |  |  |  |  |  |  |  |  |  |
|  | | White |  | 830 (65.5) |  | 64 (59.3) | 301 (60.8) | 86 (65.6) |  | 163 (62.2) | 177 (62.8) |
|  | | Non-white |  | 437 (34.5) |  | 44 (40.7) | 194 (39.2) | 45 (34.4) |  | 99 (37.8) | 105 (37.2) |
|  | Marital Status | |  |  |  |  |  |  |  |  |  |
|  | | Single |  | 74 (5.8) |  | 24 (22.2) | 92 (18.6) | 36 (27.5) |  | 53 (20.2) | 56 (19.9) |
|  | | Married |  | 713 (56.3) |  | 45 (41.7) | 180 (36.4) | 32 (24.4) |  | 91 (34.7) | 96 (34.0) |
|  | | Widowed |  | 27 (2.1) |  | 1 (0.9) | 10 (2.0) | 3 (2.3) |  | 4 (1.5) | 6 (2.1) |
|  | | Separated |  | 58 (4.6) |  | 6 (5.6) | 43 (8.7) | 17 (13.0) |  | 20 (7.6) | 25 (8.9) |
|  | | Cohabitation |  | 395 (31.1) |  | 32 (29.6) | 169 (34.1) | 43 (32.8) |  | 94 (35.9) | 99 (35.1) |
|  | | Missing |  | 0 (0.0) |  | 0 (0.0) | 1 (0.2) | 0 (0.0) |  | 0 (0.0) | 0 (0.0) |
|  | Education | |  |  |  |  |  |  |  |  |  |
|  | | <Elementary |  | 282 (22.3) |  | 20 (18.5) | 100 (20.2) | 22 (16.8) |  | 48 (18.3) | 52 (18.4) |
|  | | Elementary |  | 752 (59.4) |  | 67 (62.0) | 300 (60.6) | 75 (57.3) |  | 170 (64.9) | 172 (61.0) |
|  | | High School |  | 200 (15.8) |  | 19 (17.6) | 82 (16.6) | 31 (23.7) |  | 40 (15.3) | 47 (16.7) |
|  | | College/University |  | 31 (2.4) |  | 2 (1.9) | 13 (2.6) | 3 (2.3) |  | 4 (1.5) | 11 (3.9) |
|  | | Missing |  | 2 (0.2) |  | 0 (0.0) | 0 (0.0) | 0 (0.0) |  | 0 (0.0) | 0 (0.0) |
|  | Occupation | |  |  |  |  |  |  |  |  |  |
|  | | Domestic |  | 757 (59.7) |  | 43 (39.8) | 263 (53.1) | 69 (52.7) |  | 139 (53.1) | 149 (52.8) |
|  | | Non-Domestic |  | 453 (35.8) |  | 58 (53.7) | 213 (43.0) | 55 (42.0) |  | 114 (43.5) | 126 (44.7) |
|  | | Industrial |  | 56 (4.4) |  | 7 (6.5) | 19 (3.8) | 7 (5.3) |  | 9 (3.4) | 7 (2.5) |
|  | | Missing |  | 1 (0.1) |  | 0 (0.0) | 0 (0.0) | 0 (0.0) |  | 0 (0.0) | 0 (0.0) |
|  | Income Tertiles | |  |  |  |  |  |  |  |  |  |
|  | | Low |  | 461 (36.4) |  | 31 (28.7) | 180 (36.4) | 47 (35.9) |  | 89 (34.0) | 96 (34.0) |
|  | | Middle |  | 371 (29.3) |  | 35 (32.4) | 146 (29.5) | 35 (26.7) |  | 80 (30.5) | 82 (29.1) |
|  | | High |  | 411 (32.4) |  | 39 (36.1) | 158 (31.9) | 45 (34.4) |  | 87 (33.2) | 99 (35.1) |
|  | | Missing |  | 24 (1.9) |  | 3 (2.8) | 11 (2.2) | 4 (3.1) |  | 6 (2.3) | 5 (1.8) |
|  | Smoking | |  |  |  |  |  |  |  |  |  |
|  | | Never |  | 647 (51.1) |  | 51 (47.2) | 210 (42.4) | 60 (45.8) |  | 115 (43.9) | 127 (45.0) |
|  | | Former |  | 403 (31.8) |  | 35 (32.4) | 191 (38.6) | 49 (37.4) |  | 97 (37.0) | 104 (36.9) |
|  | | Current |  | 217 (17.1) |  | 22 (20.4) | 94 (19.0) | 22 (16.8) |  | 50 (19.1) | 51 (18.1) |
|  | | |  |  |  |  |  |  |  |  |  |
| **Sexual Behaviour and Reproductive Health Factors** | | | | |  |  |  |  |  |  |  |
|  | Age at first intercourse | |  |  |  |  |  |  |  |  |  |
|  | | ≤15 |  | 319 (25.2) |  | 27 (25.0) | 166 (33.5) | 41 (31.3) |  | 81 (30.9) | 88 (31.2) |
|  | | 16-19 |  | 571 (45.1) |  | 56 (51.9) | 245 (49.5) | 61 (46.6) |  | 130 (49.6) | 144 (51.1) |
|  | | 20+ |  | 377 (29.8) |  | 25 (23.1) | 84 (17.0) | 29 (22.1) |  | 51 (19.5) | 50 (17.7) |
|  | Lifetime number of sexual partners | |  |  |  |  |  |  |  |  |  |
|  | | 0-1 |  | 631 (49.8) |  | 38 (35.2) | 166 (33.5) | 33 (25.2) |  | 96 (36.6) | 92 (32.6) |
|  | | 2-3 |  | 404 (31.9) |  | 36 (33.3) | 203 (41.0) | 53 (40.5) |  | 104 (39.7) | 117 (41.5) |
|  | | 4+ |  | 231 (18.2) |  | 34 (31.5) | 126 (25.5) | 45 (34.4) |  | 62 (23.7) | 73 (25.9) |
|  | | Missing |  | 1 (0.1) |  | 0 (0.0) | 0 (0.0) | 0 (0.0) |  | 0 (0.0) | 0 (0.0) |
|  | Number of pregnancies | |  |  |  |  |  |  |  |  |  |
|  | | 0-1 |  | 190 (15.0) |  | 22 (20.4) | 98 (19.8) | 25 (19.1) |  | 49 (18.7) | 62 (22.0) |
|  | | 2-6 |  | 947 (74.7) |  | 77 (71.3) | 342 (69.1) | 90 (68.7) |  | 188 (71.8) | 190 (67.4) |
|  | | 7+ |  | 125 (9.9) |  | 8 (7.4) | 53 (10.7) | 15 (11.5) |  | 23 (8.8) | 27 (9.6) |
|  | | Missing |  | 5 (0.4) |  | 1 (0.9) | 2 (0.4) | 1 (0.8) |  | 2 (0.8) | 3 (1.1) |
|  | Previous Pap smears | |  |  |  |  |  |  |  |  |  |
|  | | 0 |  | 19 (1.5) |  | 7 (6.5) | 20 (4.0) | 6 (4.6) |  | 11 (4.2) | 11 (3.9) |
|  | | 1-4 |  | 509 (40.2) |  | 42 (38.9) | 236 (47.7) | 62 (47.3) |  | 129 (49.2) | 148 (52.5) |
|  | | 5+ |  | 712 (56.2) |  | 56 (51.9) | 225 (45.5) | 60 (45.8) |  | 117 (44.7) | 118 (41.8) |
|  | | Missing |  | 27 (2.1) |  | 3 (2.8) | 14 (2.8) | 3 (2.3) |  | 5 (1.9) | 5 (1.8) |
|  | Condom | |  |  |  |  |  |  |  |  |  |
|  | | Never |  | 449 (35.4) |  | 28 (25.9) | 159 (32.1) | 29 (22.1) |  | 66 (25.2) | 84 (29.8) |
|  | | Former |  | 566 (44.7) |  | 51 (47.2) | 211 (42.6) | 69 (52.7) |  | 125 (47.7) | 123 (43.6) |
|  | | Current |  | 250 (19.7) |  | 29 (26.9) | 123 (24.9) | 33 (25.2) |  | 69 (26.3) | 73 (25.9) |
|  | | Missing |  | 2 (0.2) |  | 0 (0.0) | 2 (0.4) | 0 (0.0) |  | 2 (0.8) | 2 (0.7) |
|  | Oral Contraceptives | |  |  |  |  |  |  |  |  |  |
|  | | Never |  | 181 (14.3) |  | 18 (16.7) | 83 (16.8) | 18 (13.7) |  | 42 (16.0) | 41 (14.5) |
|  | | <6 years |  | 690 (54.5) |  | 62 (57.4) | 285 (57.6) | 92 (70.2) |  | 165 (63.0) | 164 (58.2) |
|  | | 6+ years |  | 396 (31.3) |  | 28 (25.9) | 127 (25.7) | 21 (16.0) |  | 55 (21.0) | 77 (27.3) |
|  | Intrauterine Device | |  |  |  |  |  |  |  |  |  |
|  | | No |  | 749 (59.1) |  | 82 (75.9) | 321 (64.9) | 89 (67.9) |  | 179 (68.3) | 187 (66.3) |
|  | | Yes |  | 518 (40.9) |  | 26 (24.1) | 174 (35.2) | 42 (32.1) |  | 83 (31.7) | 95 (33.7) |
|  | Tubal Sterilization | |  |  |  |  |  |  |  |  |  |
|  | | No |  | 1076 (84.9) |  | 93 (86.1) | 444 (89.7) | 119 (90.8) |  | 239 (91.2) | 254 (90.1) |
|  | | Yes |  | 191 (15.1) |  | 15 (13.9) | 51 (10.3) | 12 (9.2) |  | 23 (8.8) | 28 (9.9) |
|  | Birth Control Injection | |  |  |  |  |  |  |  |  |  |
|  | | No |  | 1102 (87.0) |  | 82 (75.9) | 411 (83.0) | 108 (82.4) |  | 213 (81.3) | 236 (83.7) |
|  | | Yes |  | 165 (13.0) |  | 26 (24.1) | 84 (17.0) | 23 (17.6) |  | 49 (18.7) | 46 (16.3) |
|  | Natural Products | |  |  |  |  |  |  |  |  |  |
|  | | No |  | 1233 (97.3) |  | 108 (100.0) | 491 (99.2) | 129 (98.5) |  | 261 (99.6) | 279 (98.9) |
|  | | Yes |  | 34 (2.7) |  | 0 (0.0) | 4 (0.8) | 2 (1.5) |  | 1 (0.4) | 3 (1.1) |
|  | Vaginal Products | |  |  |  |  |  |  |  |  |  |
|  | | No |  | 1229 (97.0) |  | 108 (100.0) | 487 (98.4) | 258 (98.5) |  | 278 (98.6) | 229 (98.3) |
|  | | Yes |  | 38 (3.0) |  | 0 (0.0) | 8 (1.6) | 4 (1.5) |  | 4 (1.4) | 4 (1.7) |
|  | | |  |  |  |  |  |  |  |  |  |
| **Genital Health and Hygiene Factors** | | |  |  |  |  |  |  |  |  |  |
|  | Menstrual Cloth | |  |  |  |  |  |  |  |  |  |
|  | | No |  | 782 (61.7) |  | 84 (77.8) | 329 (66.5) | 86 (65.7) |  | 176 (67.2) | 203 (72.0) |
|  | | Yes |  | 484 (38.2) |  | 24 (22.2) | 164 (33.1) | 44 (33.6) |  | 85 (32.4) | 79 (28.0) |
|  | | Missing |  | 1 (0.1) |  | 0 (0.0) | 2 (0.4) | 1 (0.8) |  | 1 (0.4) | 0 (0.0) |
|  | Hygienic Tampon | |  |  |  |  |  |  |  |  |  |
|  | | No |  | 1124 (88.7) |  | 88 (81.5) | 416 (84.0) | 108 (82.4) |  | 218 (83.2) | 234 (83.0) |
|  | | Yes |  | 142 (11.2) |  | 20 (18.5) | 77 (15.6) | 22 (16.8) |  | 43 (16.4) | 48 (17.0) |
|  | | Missing |  | 1 (0.1) |  | 0 (0.0) | 2 (0.4) | 1 (0.8) |  | 1 (0.4) | 0 (0.0) |
|  | Douching | |  |  |  |  |  |  |  |  |  |
|  | | Never/Occasional |  | 1191 (94.0) |  | 99 (91.7) | 456 (92.1) | 129 (98.5) |  | 242 (92.4) | 259 (91.8) |
|  | | Frequent |  | 72 (5.8) |  | 9 (8.3) | 37 (7.5) | 2 (1.5) |  | 18 (6.9) | 21 (7.5) |
|  | | Missing |  | 2 (0.2) |  | 0 (0.0) | 2 (0.4) | 0 (0.0) |  | 2 (0.8) | 2 (0.7) |
|  | Douching Products Used | |  |  |  |  |  |  |  |  |  |
|  | | None |  | 841 (69.1) |  | 74 (71.2) | 352 (73.3) | 92 (74.8) |  | 192 (75.0) | 204 (75.0) |
|  | | Natural |  | 377 (29.8) |  | 30 (27.8) | 128 (25.9) | 31 (23.7) |  | 64 (24.4) | 68 (24.1) |
|  | | Medical |  | 31 (2.5) |  | 2 (1.9) | 11 (2.2) | 5 (3.8) |  | 4 (1.5) | 8 (2.8) |
|  | | Unknown |  | 33 (2.6) |  | 2 (1.9) | 7 (1.4) | 7 (5.3) |  | 3 (1.2) | 5 (1.8) |
|  | Genital Discomfort | |  |  |  |  |  |  |  |  |  |
|  | | No |  | 806 (63.6) |  | 68 (63.0) | 319 (64.4) | 85 (64.9) |  | 171 (65.3) | 181 (64.2) |
|  | | Yes |  | 431 (36.4) |  | 40 (37.0) | 176 (35.6) | 46 (35.1) |  | 91 (34.7) | 101 (35.8) |
|  | Recent Discomfort | |  |  |  |  |  |  |  |  |  |
|  | | No |  | 840 (66.3) |  | 71 (65.7) | 314 (63.4) | 80 (61.1) |  | 165 (63.0) | 172 (61.0) |
|  | | Yes |  | 427 (33.7) |  | 37 (34.3) | 181 (36.6) | 51 (38.9) |  | 97 (37.0) | 110 (39.0) |
|  | Pain or Bleeding | |  |  |  |  |  |  |  |  |  |
|  | | No |  | 1257 (99.2) |  | 106 (98.2) | 491 (99.2) | 130 (99.2) |  | 261 (99.6) | 279 (98.9) |
|  | | Yes |  | 10 (0.8) |  | 2 (1.9) | 4 (0.8) | 1 (0.8) |  | 1 (0.4) | 3 (1.1) |
|  | | |  |  |  |  |  |  |  |  |  |
| **Gynecologic Products** | | |  |  |  |  |  |  |  |  |  |
|  | Antibiotic | |  |  |  |  |  |  |  |  |  |
|  | | Never |  | 114 (9.0) |  | 5 (4.6) | 48 (9.7) | 9 (6.9) |  | 20 (7.6) | 21 (7.5) |
|  | | Former |  | 311 (24.6) |  | 25 (23.2) | 108 (21.8) | 32 (24.4) |  | 54 (20.6) | 65 (23.1) |
|  | | Current |  | 70 (5.5) |  | 9 (8.3) | 31 (6.3) | 8 (6.1) |  | 12 (4.6) | 27 (9.6) |
|  | Antifungal | |  |  |  |  |  |  |  |  |  |
|  | | Never |  | 114 (9.0) |  | 5 (4.6) | 48 (9.7) | 9 (6.9) |  | 20 (7.6) | 21 (7.5) |
|  | | Former |  | 339 (26.8) |  | 37 (34.3) | 137 (27.7) | 38 (29.0) |  | 83 (31.7) | 72 (25.5) |
|  | | Current |  | 65 (5.1) |  | 2 (1.9) | 25 (5.1) | 6 (4.6) |  | 14 (5.3) | 14 (5.0) |
|  | Abrasion | |  |  |  |  |  |  |  |  |  |
|  | | Never |  | 114 (9.0) |  | 5 (4.6) | 48 (9.7) | 9 (6.9) |  | 20 (7.6) | 21 (7.5) |
|  | | Former |  | 9 (0.7) |  | 2 (1.9) | 4 (0.8) | 3 (2.3) |  | 83 (31.7) | 4 (1.4) |
|  | | Current |  | 3 (0.2) |  | 0 (0.0) | 1 (0.2) | 0 (0.0) |  | 14 (5.3) | 0 (0.0) |
|  | External Products | |  |  |  |  |  |  |  |  |  |
|  | | Never |  | 114 (9.0) |  | 5 (4.6) | 48 (9.7) | 9 (6.9) |  | 20 (7.6) | 21 (7.5) |
|  | | Former |  | 9 (0.7) |  | 1 (0.9) | 3 (0.6) | 0 (0.0) |  | 2 (0.8) | 1 (0.4) |
|  | | Current |  | 11 (0.9) |  | 1 (0.9) | 6 (1.2) | 0 (0.0) |  | 1 (0.4) | 2 (0.7) |
|  | Unknown | |  |  |  |  |  |  |  |  |  |
|  | | Never |  | 114 (9.0) |  | 5 (4.6) | 48 (9.7) | 9 (6.9) |  | 20 (7.6) | 21 (7.5) |
|  | | Former |  | 354 (27.9) |  | 22 (20.4) | 120 (24.2) | 31 (23.7) |  | 54 (20.6) | 63 (22.3) |
|  | | Current |  | 67 (5.3) |  | 7 (6.5) | 27 (5.5) | 9 (6.9) |  | 16 (6.1) | 20 (7.1) |
|  | Homemade Gynecologic Products | |  |  |  |  |  |  |  |  |  |
|  | | None |  | 786 (62.0) |  | 68 (63.0) | 326 (65.9) | 83 (63.4) |  | 173 (66.0) | 191 (67.7) |
|  | | Natural |  | 432 (34.1) |  | 36 (33.3) | 143 (28.9) | 40 (30.5) |  | 78 (29.8) | 81 (28.7) |
|  | | Medical |  | 62 (4.9) |  | 4 (3.7) | 28 (5.7) | 5 (3.8) |  | 11 (4.2) | 11 (3.9) |
|  | | Unknown |  | 13 (1.0) |  | 2 (1.9) | 5 (1.0) | 5 (3.8) |  | 2 (0.8) | 5 (1.8) |
|  | | |  |  |  |  |  |  |  |  |  |
| **Vaginal Health Characteristics** | | |  |  |  |  |  |  |  |  |  |
|  | Cervical Ectropion | |  |  |  |  |  |  |  |  |  |
|  | | No |  | 1239 (97.8) |  | 107 (99.1) | 486 (98.2) | 128 (97.7) |  | 255 (97.3) | 277 (98.2) |
|  | | Yes |  | 28 (2.2) |  | 1 (0.9) | 9 (1.8) | 3 (2.3) |  | 7 (2.7) | 5 (1.8) |
|  | Gynecologic Surgery | |  |  |  |  |  |  |  |  |  |
|  | | No |  | 1173 (92.6) |  | 94 (87.0) | 463 (93.5) | 122 (93.1) |  | 242 (92.4) | 258 (91.5) |
|  | | Yes |  | 94 (7.4) |  | 14 (13.0) | 32 (6.5) | 9 (6.9) |  | 20 (7.6) | 24 (8.5) |
|  | Gynecologic Infection/ Treatment | |  |  |  |  |  |  |  |  |  |
|  | | No |  | 948 (74.8) |  | 79 (73.2) | 379 (76.6) | 100 (76.3) |  | 203 (77.5) | 208 (73.8) |
|  | | Yes |  | 319 (25.2) |  | 29 (26.9) | 116 (23.4) | 31 (23.7) |  | 59 (22.5) | 74 (26.2) |
|  | |  |  |  |  |  |  |  |  |  |  |
|  | Previous Gynecologic Infections | |  |  |  |  |  |  |  |  |  |
|  | | None |  | 976 (77.0) |  | 71 (65.7) | 368 (74.3) | 95 (72.5) |  | 193 (73.7) | 207 (73.4) |
|  | | HPV-related |  | 38 (3.0) |  | 15 (13.9) | 36 (7.3) | 7 (5.3) |  | 27 (10.3) | 21 (7.5) |
|  | | Other |  | 247 (19.5) |  | 22 (20.4) | 90 (18.2) | 29 (22.1) |  | 42 (16.0) | 54 (19.2) |
|  | | Missing |  | 6 (0.5) |  | 0 (0.0) | 1 (0.2) | 0 (0.0) |  | 0 (0.0) | 0 (0.0) |
|  | | |  |  |  |  |  |  |  |  |  |
| **Cytology Observations** | | |  |  |  |  |  |  |  |  |  |
|  | Bacterial Infection | |  |  |  |  |  |  |  |  |  |
|  | | No |  | 1187 (93.7) |  | 99 (91.7) | 451 (91.1) | 120 (91.6) |  | 242 (92.4) | 255 (90.4) |
|  | | Yes |  | 80 (6.3) |  | 9 (8.3) | 44 (8.9) | 11 (8.4) |  | 20 (7.6) | 27 (9.6) |
|  | Fungal Infection | |  |  |  |  |  |  |  |  |  |
|  | | No |  | 1177 (92.9) |  | 96 (88.9) | 449 (90.7) | 117 (89.3) |  | 242 (92.4) | 257 (91.1) |
|  | | Yes |  | 90 (7.1) |  | 12 (11.1) | 46 (9.3) | 14 (10.7) |  | 20 (7.6) | 25 (8.9) |
|  | Inflammation | |  |  |  |  |  |  |  |  |  |
|  | | No |  | 992 (78.3) |  | 69 (63.9) | 344 (69.5) | 94 (71.8) |  | 179 (68.3) | 199 (70.6) |
|  | | Yes |  | 275 (21.7) |  | 39 (36.1) | 151 (30.5) | 37 (28.2) |  | 83 (31.7) | 83 (29.4) |

^*^ Subgenus 1 (HPVs-6, 11, 32, 40, 42, 44, 54 and 55), Subgenus 2 (HPVs-16, 18, 26 31, 33-35, 39, 45, 51-53, 56, 58, 59, 66-70, 73 and 82) and Subgenus 3 (HPVs-57, 61, 62, 71, 72, 81, 83, 84 and 89) infections were determined based on the phylogenetic classification of HPV types.

^†^Transient HPV infections were defined as one positive test result in the first four visits, followed by two subsequent negative results. Persistent HPV infections were defined as two or more positive HPV test results over the first four visits, with no more than one negative result between two positive test results.

**Table S3.** Crude and restricted crude (Crude^R^) odds ratios of associations between reproductive health and vaginal hygiene and the one-year period prevalence of HPV infection according to phylogenetic group in the Ludwig-McGill Cohort Study

| **Variable** | | |  | **Subgenus** **1^*^** | |  | **Subgenus** **2^*^** | | |  | **Subgenus** **3^*^** | | |
| --- | --- | --- | --- | --- | --- | --- | --- | --- | --- | --- | --- | --- | --- |
|  |  |  |  | **Crude**  **OR^†^ (95%CI)** | **Restricted Crude**  **OR^‡^ (95%CI)** |  | **Crude**  **OR^†^ (95%CI)** | | **Restricted Crude**  **OR^‡^ (95%CI)** |  | **Crude**  **OR^†^ (95%CI)** | | **Restricted Crude**  **OR^‡^ (95%CI)** |
| **Reproductive Health Factors** | | |  |  |  |  |  | |  |  |  | |  |
|  | Condom Use | |  |  |  |  |  | |  |  |  | |  |
|  | | Former vs. Never |  | 1.41 (0.88, 2.27) | 1.75 (0.82, 3.72) |  | 1.01 (0.80, 1.29) | | 0.95 (0.73, 1.23) |  | 1.89 (1.21, 2.95) | | 1.59 (0.83, 3.05) |
|  | | Current vs. Never |  | 1.69 (0.99, 2.89) | 1.62 (0.65, 4.03) |  | 1.33 (1.01, 1.75) | | 1.28 (0.94, 1.74) |  | 1.88 (1.12, 3.14) | | 1.92 (0.91, 4.05) |
|  | Oral Contraceptives | |  |  |  |  |  | |  |  |  | |  |
|  | | <6 years vs. Never |  | 0.92 (0.54, 1.59) | 1.26 (0.47, 3.35) |  | 0.89 (0.67, 1.20) | | 0.84 (0.61, 1.16) |  | 1.41 (0.84, 2.38) | | 0.88 (0.44, 1.76) |
|  | | 6+ years vs. Never |  | 0.79 (0.43, 1.45) | 1.10 (0.38, 3.16) |  | 0.72 (0.52, 1.00) | | 0.72 (0.51, 1.03) |  | 0.58 (0.30, 1.11) | | 0.37 (0.15, 0.92) |
|  | Intrauterine Device | |  |  |  |  |  | |  |  |  | |  |
|  | | Yes vs. No |  | 0.49 (0.31, 0.76) | 0.35 (0.16, 0.77) |  | 0.82 (0.66, 1.01) | | 0.83 (0.65, 1.05) |  | 0.73 (0.50, 1.07) | | 0.84 (0.49, 1.46) |
|  | Tubal Sterilization | |  |  |  |  |  | |  |  |  | |  |
|  | | Yes vs. No |  | 1.02 (0.58, 1.78) | 1.37 (0.62, 3.00) |  | 0.65 (0.47, 0.91) | | 0.65 (0.45, 0.94) |  | 0.62 (0.34, 1.13) | | 0.54 (0.21, 1.37) |
|  | Contraceptive Injection | |  |  |  |  |  | |  |  |  | |  |
|  | | Yes vs. No |  | 2.00 (1.26, 3.17) | 2.15 (1.04, 4.48) |  | 1.33 (1.00, 1.76) | | 1.17 (0.84, 1.62) |  | 1.30 (0.81, 2.08) | | 1.09 (0.51, 2.34) |
|  | Natural Products | |  |  |  |  |  | |  |  |  | |  |
|  | | Yes vs. No |  | - | - |  | 0.30 (0.11, 0.85) | | 0.39 (0.14, 1.10) |  | 0.69 (0.17, 2.90) | | 1.32 (0.31, 5.63) |
|  | Vaginal Products | |  |  |  |  |  | |  |  |  | |  |
|  | | Yes vs. No |  | - | - |  | 0.56 (0.26, 1.21) | | 0.70 (0.32, 1.52) |  | 0.28 (0.04, 2.07) | | 0.58 (0.08, 4.28) |
| **Genital Health and Hygiene Factors** | | | | |  |  |  | |  |  |  | |  |
|  | Menstrual Cloth | |  |  |  |  |  | |  |  |  | |  |
|  | | Yes vs. No |  | 0.48 (0.30, 0.76) | 0.52 (0.25, 1.07) |  | 0.82 (0.66, 1.02) | | 0.86 (0.68, 1.10) |  | 0.88 (0.60, 1.28) | | 1.18 (0.69, 2.01) |
|  | Hygienic Tampon | |  |  |  |  |  | |  |  |  | |  |
|  | | Yes vs. No |  | 1.63 (0.98, 2.70) | 1.36 (0.56, 3.28) |  | 1.42 (1.06, 1.91) | | 1.39 (0.99, 1.93) |  | 1.45 (0.90, 2.35) | | 1.29 (0.60, 2.78) |
|  | Douching | |  |  |  |  |  | |  |  |  | |  |
|  | | Frequent vs. Infrequent |  | 1.40 (0.69, 2.85) | 0.74 (0.60, 5.01) |  | 1.33 (0.88, 1.99) | | 1.45 (0.94, 2.24) |  | 0.22 (0.05, 0.90) | | 0.29 (0.04, 2.11) |
|  | Douching Products | |  |  |  |  | |  |  |  | |  |  |
|  | | Natural vs. None |  | 0.95 (0.62, 1.48) | 1.43 (0.75, 2.71) |  | | 0.81 (0.64, 1.03) | 0.81 (0.62, 1.05) |  | | 0.78 (0.51, 1.19) | 0.80 (0.43, 1.49) |
|  | | Medical vs. None |  | 0.75 (0.18, 3.15) | - |  | | 0.84 (0.42, 1.67) | 0.80 (0.37, 1.77) |  | | 1.59 (0.61, 4.13) | 1.39 (0.32, 6.02) |
|  | | Unknown vs. None |  | 0.75 (0.18, 3.15) | - |  | | 0.48 (0.21, 1.08) | 0.28 (0.09, 0.93) |  | | 2.34 (1.02, 5.40) | 3.27 (1.21, 8.83) |
|  | Genital Discomfort | |  |  |  |  | |  |  |  | |  |  |
|  | | Yes vs. No |  | 1.05 (0.70, 1.56) | 1.01 (0.53, 1.92) |  | | 0.97 (0.78, 1.20) | 0.93 (0.73, 1.18) |  | | 0.96 (0.66, 1.39) | 0.81 (0.46, 1.43) |
|  | Recent Discomfort | |  |  |  |  | |  |  |  | |  |  |
|  | | Yes vs. No |  | 1.00 (0.66, 1.50) | 0.72 (0.36, 1.45) |  | | 1.14 (0.92, 1.42) | 1.08 (0.85, 1.38) |  | | 1.24 (0.86, 1.78) | 0.84 (0.47, 1.49) |
|  | Pain or Bleeding | |  |  |  |  | |  |  |  | |  |  |
|  | | Yes vs. No |  | 2.35 (0.53, 10.48) | 3.14 (0.39, 25.14) |  | | 0.92 (0.30, 2.88) | 1.01 (0.28, 3.68) |  | | 0.88 (0.12, 6.73) | 2.24 (0.28, 17.84) |
| **Gynecologic Products** | | |  |  |  |  | |  |  |  | |  |  |
|  | Antibiotic | |  |  |  |  | |  |  |  | |  |  |
|  | | Former vs. Never |  | 1.95 (0.74, 5.19) | - |  | | 0.81 (0.54, 1.20) | 0.68 (0.44, 1.05) |  | | 1.38 (0.64, 2.95) | 0.95 (0.33, 2.73) |
|  | | Current vs. Never |  | 2.90 (0.95, 8.91) | - |  | | 0.98 (0.58, 1.67) | 0.89 (0.49, 1.61) |  | | 1.39 (0.52, 3.71) | 1.31 (0.34, 5.02) |
|  | Antifungal | |  |  |  |  | |  |  |  | |  |  |
|  | | Former vs. Never |  | 2.56 (0.99, 6.61) | - |  | | 0.92 (0.62, 1.36) | 0.82 (0.54, 1.24) |  | | 1.43 (0.67, 3.01) | 0.94 (0.33, 2.67) |
|  | | Current vs. Never |  | 0.72 (0.14, 3.77) | - |  | | 0.92 (0.52, 1.62) | 0.84 (0.45, 1.54) |  | | 1.22 (0.42, 3.54) | 1.05 (0.24, 4.55) |
|  | Abrasion | |  |  |  |  | |  |  |  | |  |  |
|  | | Former vs. Never |  | 4.66 (0.83, 26.22) | - |  | | 0.83 (0.26, 2.71) | 0.60 (0.13, 2.91) |  | | 4.08 (0.98, 16.93) | 5.07 (0.86, 29.89) |
|  | | Current vs. Never |  | - | - |  | | 0.83 (0.08, 8.21) | 0.90 (0.09, 8.94) |  | | - | - |
|  | External Products | |  |  |  |  | |  |  |  | |  |  |
|  | | Former vs. Never |  | 2.72 (0.29, 25.15) | - |  | | 0.75 (0.20, 2.84) | 0.90 (0.23, 3.50) |  | | - | - |
|  | | Current vs. Never |  | 2.04 (0.22, 18.53) | - |  | | 1.36 (0.48, 3.89) | 1.23 (0.40, 3.76) |  | | - | - |
|  | Unknown Products | |  |  |  |  | |  |  |  | |  |  |
|  | | Former vs. Never |  | 1.50 (0.56, 4.03) | - |  | | 0.79 (0.53, 1.17) | 0.74 (0.49, 1.13) |  | | 1.17 (0.54, 2.51) | 0.97 (0.34, 2.72) |
|  | | Current vs. Never |  | 2.40 (0.74, 7.78) | - |  | | 0.90 (0.52, 1.56) | 0.81 (0.44, 1.49) |  | | 1.71 (0.66, 4.46) | 1.36 (0.35, 5.25) |
|  | Home-made Products | |  |  |  |  | |  |  |  | |  |  |
|  | | Natural vs. None |  | 1.03 (0.68, 1.56) | 1.16 (0.61, 2.20) |  | | 0.81 (0.64, 1.01) | 0.73 (0.57, 0.95) |  | | 0.93 (0.63, 1.38) | 0.65 (0.35, 1.22) |
|  | | Medical vs. None |  | 0.74 (0.26, 2.06) | - |  | | 1.13 (0.71, 1.79) | 1.09 (0.66, 1.81) |  | | 0.75 (0.30, 1.90) | 0.98 (0.29, 3.25) |
|  | | Unknown vs. None |  | 1.72 (0.40, 7.50) | - |  | | 0.82 (0.30, 2.26) | 0.24 (0.03, 1.81) |  | | 4.13 (1.48, 11.56) | 4.65 (1.27, 17.00) |
| **Vaginal Health Characteristics** | | |  |  |  |  | |  |  |  | |  |  |
|  | Cervical Ectropion | |  |  |  |  | |  |  |  | |  |  |
|  | | Yes vs. No |  | 0.41 (0.06, 3.03) | 1.11 (0.15, 8.33) |  | | 0.80 (0.38, 1.69) | 0.96 (0.43, 2.12) |  | | 1.08 (0.33, 3.54) | 1.61 (0.37, 6.93) |
|  | Gynecologic Surgery | |  |  |  |  | |  |  |  | |  |  |
|  | | Yes vs. No |  | 1.88 (1.04, 3.39) | 3.51 (1.63, 7.57) |  | | 0.79 (0.52, 1.18) | 0.96 (0.62, 1.50) |  | | 0.88 (0.44, 1.78) | 1.75 (0.77, 3.96) |
|  | Gynecologic Treatment/Infection | |  |  |  |  | |  |  |  | |  |  |
|  | | Yes vs. No |  | 1.72 (1.14, 2.59) | 1.23 (0.62, 2.44) |  | | 1.12 (0.89, 1.43) | 0.97 (0.66, 1.14) |  | | 1.22 (0.82, 1.82) | 0.97 (0.52, 1.79) |
|  | Previous Gynecologic Infection | |  |  |  |  | |  |  |  | |  |  |
|  | | HPV vs. None |  | 4.24 (2.31, 7.79) | 4.94 (1.80, 13.57) |  | | 2.23 (1.42, 3.50) | 2.18 (1.28, 3.69) |  | | 1.30 (0.58, 2.89) | 1.22 (0.29, 5.24) |
|  | | Non-HPV vs. None |  | 1.22 (0.75, 2.00) | 1.52 (0.72, 3.19) |  | | 0.94 (0.72, 1.22) | 0.96 (0.72, 1.30) |  | | 1.21 (0.78, 1.86) | 1.22 (0.65, 2.31) |
| **Cytology Observations** | | |  |  |  |  | |  |  |  | |  |  |
|  | Bacterial Infection | |  |  |  |  | |  |  |  | |  |  |
|  | | Yes vs. No |  | 1.22 (0.60, 2.47) | 1.18 (0.36, 3.92) |  | | 1.42 (0.97, 2.08) | 1.30 (0.84, 2.02) |  | | 1.26 (0.66, 2.41) | 1.14 (0.40, 3.25) |
|  | Fungal Infection | |  |  |  |  | |  |  |  | |  |  |
|  | | Yes vs. No |  | 1.51 (0.81, 2.82) | 1.04 (0.31, 3.44) |  | | 1.28 (0.89, 1.86) | 1.23 (0.81, 1.88) |  | | 1.49 (0.83, 2.66) | 1.57 (0.66, 3.77) |
|  | General Inflammation | |  |  |  |  | |  |  |  | |  |  |
|  | | Yes vs. No |  | 1.82 (1.21, 2.74) | 1.92 (0.99, 3.73) |  | | 1.54 (1.22, 1.94) | 1.53 (1.18, 1.99) |  | | 1.22 (0.82, 1.83) | 0.99 (0.52, 1.91) |

^*^ Subgenus 1 (HPVs-6, 11, 32, 40, 42, 44, 54 and 55), Subgenus 2 (HPVs-16, 18, 26 31, 33-35, 39, 45, 51-53, 56, 58, 59, 66-70, 73 and 82) and Subgenus 3 (HPVs-57, 61, 62, 71, 72, 81, 83, 84 and 89) infections were determined based on the phylogenetic classification of HPV types.

^†^Crude OR compares women with a specific group infection against a floating referent group of all those who did not have that particular group infection.

^‡^Crude^R^ OR compares women with a specific group infection against a fixed referent group of HPV-negative women.
